# Antiplatelet Effect of Catechol Is Related to Inhibition of Cyclooxygenase, Reactive Oxygen Species, ERK/p38 Signaling and Thromboxane A_2_ Production

**DOI:** 10.1371/journal.pone.0104310

**Published:** 2014-08-14

**Authors:** Mei-Chi Chang, Hsiao-Hua Chang, Tong-Mei Wang, Chiu-Po Chan, Bor-Ru Lin, Sin-Yuet Yeung, Chien-Yang Yeh, Ru-Hsiu Cheng, Jiiang-Huei Jeng

**Affiliations:** 1 Biomedical Science Team, Chang Gung University of Science and Technology, Taoyuan,Taiwan; 2 Laboratory of Pharmacology & Toxicology, Graduate Institute of Clinical Dentistry and Department of Dentistry, National Taiwan University Hospital and National Taiwan University Medical College, Taipei, Taiwan; 3 Department of Dentistry, Chang Gung Memorial Hospital and Chang Gung University, Taipei, Taiwan; 4 Department of Diagnotherapeutics, National Taiwan University Hospital, Taipei, Taiwan; The University of Hong Kong, China

## Abstract

Catechol (benzenediol) is present in plant-derived products, such as vegetables, fruits, coffee, tea, wine, areca nut and cigarette smoke. Because platelet dysfunction is a risk factor of cardiovascular diseases, including stroke, atherosclerosis and myocardial infarction, the purpose of this study was to evaluate the anti-platelet and anti-inflammatory effect of catechol and its mechanisms. The effects of catechol on cyclooxygenase (COX) activity, arachidonic acid (AA)-induced aggregation, thromboxane B_2_ (TXB_2_) production, lactate dehydrogenase (LDH) release, reactive oxygen species (ROS) production and extracellular signal-regulated kinase (ERK)/p38 phosphorylation were determined in rabbit platelets. In addition, its effect on IL-1β-induced prostaglandin E_2_ (PGE_2_) production by fibroblasts was determined. The *ex vivo* effect of catechol on platelet aggregation was also measured. Catechol (5-25 µM) suppressed AA-induced platelet aggregation and inhibited TXB_2_ production at concentrations of 0.5–5 µM; however, it showed little cytotoxicity and did not alter U46619-induced platelet aggregation. Catechol (10–50 µM) suppressed COX-1 activity by 29–44% and COX-2 activity by 29–50%. It also inhibited IL-1β-induced PGE_2_ production, but not COX-2 expression of fibroblasts. Moreover, catechol (1–10 µM) attenuated AA-induced ROS production in platelets and phorbol myristate acetate (PMA)-induced ROS production in human polymorphonuclear leukocytes. Exposure of platelets to catechol decreased AA-induced ERK and p38 phosphorylation. Finally, intravenous administration of catechol (2.5–5 µmole/mouse) attenuated *ex vivo* AA-induced platelet aggregation. These results suggest that catechol exhibited anti-platelet and anti-inflammatory effects, which were mediated by inhibition of COX, ROS and TXA_2_ production as well as ERK/p38 phosphorylation. The anti-platelet effect of catechol was confirmed by *ex vivo* analysis. Exposure to catechol may affect platelet function and thus cardiovascular health.

## Introduction

Various benzenediols (e.g., catechol, resorcinol, hydroquinone [HQ]) are present in many plant-derived products, including vegetables, areca nut, fruits, grains, coffee, tea, beer, and wine [1]–[3]. Many catechol (i.e., pyrocatechol) derivatives have been suggested to have therapeutic potential. Phenol and catechol (1,2-benzenediol) are two major metabolites identified in the urine of workers occupationally exposed to benzene. In addition, catechol and HQ (e.g., 1,4-benzenediol) levels in peripheral blood may be a marker of exposure to benzene or cigarette smoke [4]. However, the effect of catechol on human health remains to be investigated.

Catechol scavenges diphenylpicrylhydrazyl radicals and reactive oxygen species (ROS) [5]. The structure of catechin and hydroxychavicol may be responsible for its ROS scavenging and anti-platelet activities [6]. However, Lee and Lin [7] found that catechol, pyrogallol and 1,2,4-benzenetriol may gerenate ROS and exhibit mutagenicity. In addition, catechol and HQ may antagonize transforming growth factor-β (TGF-β)-induced elimination of transformed cells suggesting a co-carcinogenic effect [8].

In many countries, cardiovascular diseases, such as atherosclerosis, stroke and myocardial infarction, are responsible for a great proportion of human morbidity and mortality [9]. Platelets play important roles in thrombosis and hemostasis through platelet adhesion, activation and aggregation; their aggregation may be initiated by various agonists, including adenosine diphosphate (ADP), thrombin, collagen, and prostaglandin endoperoxides. In addition, secretion of the contents of intracellular granules may accompany platelet aggregation, which may further recruit additional platelets to induce full aggregation and subsequent gross thrombus formation. Aberrant platelet activation may directly or indirectly induce blood clot formation, thrombosis and sustained vascular wall inflammation, resulting in cardiovascular diseases (e.g. atherosclerosis and cardiovascular attack) [10], [11]. Although catechol has been shown to inhibit arachidonic acid (AA)-induced platelet aggregation [12], [13], limited mechanistic information is available about its possible anti-platelet and anti-thrombotic effects or toxicity in cardiovascular cells.

Considering the frequency that humans are exposed catechol (pyrocatechol) along with its potential pharmacological or toxicological effects, the anti-platelet and anti-inflammatory effects of catechol and related signaling mechanisms (e.g., cyclooxygenase [COX] inhibition, thromboxane, ROS, ERK/p38 phosphorylation, etc.) were assessed in this study to determine its effects on cardiovascular health.

## Materials and Methods

### Materials

Catechol (pyrocatechol), arachidonic acid (AA), U46619 (a thromboxane receptor agonist), 2′,7′-diacethyl-chlorofluorescein (DCFH-DA), lactate dehydrogenase (LDH) assay kits, superoxide dismutase (SOD), N-acetyl-L-cysteine (NAC) and sodium citrate were obtained from Sigma (St. Louis, MO, USA). Thromboxane B_2_ (TXB_2_) ELISA kits, prostaglandin E_2_ (PGE_2_) ELISA kits and a COX inhibitor screening assay were purchased from Cayman Chemical Company (Ann Arbor, MI, USA). Primary antibodies against COX-2, GAPDH (sc-32233) and phospho-extracellular signal-regulated protein kinase (p-ERK) (sc-7383) were purchased from Santa Cruz Biotechnology (Santa Cruz, CA, USA). The p-p38 antibody was obtained from Cell Signaling Technology (Beverly, MA, USA).

### Platelet aggregation assay

Washed rabbit platelets were isolated from animals in the Animal Center of National Taiwan University Hospital as previously described [14]–[16] and were suspended in Tyrodes solution containing 1 mM calcium and 0.35% bovine serum albumin (BSA). Catechol (final concentration of 0.5–100 µM) was added to the platelets for 3 min after which AA (100 µM) or U46619 (1 µM, a TXA_2_ receptor agonist) was added, and platelet aggregation was measured using the turbidimetric method described by Born and Cross [17] using an aggregometer (Model 600B, Payton Associates, ON, Canada). The percentage of aggregation inhibition was calculated as described by Teng et al. [18]. All animal studies were approved by the Ethics Committee of the Chang Gung University of Science and Technology and National Taiwan University Hospital.

### Lactate dehydrogenase (LDH) activity assay

Following exposure of platelets to catechol (1–200 µM) for 9 min, LDH activity in the supernatant and total cell lysate was determined as an index of platelet damage using LDH activity assay kits [6], [16]. The measurement was dependent on the spectrophotometric method as described by Wroblewski and La Due [19]. LDH generated by injured platelets was compared (in a percentage basis) with the total LDH activity of platelets dissolved in 0.1% Triton X-100 (v/v).

### Thromboxane B_2_ (TXB_2_) assay

Platelets were preincubated with catechol (0.5–100 µM) for 3 min and then treated with or without AA (100 µM). EDTA (2 mM) and indomethacin (50 µM) were then added to the platelet suspension. After centrifugation at 14,000 rpm for 2 min, TXB_2_ levels were measured with Cayman ELISA kits according to the manufacturer's instructions [6], [15].

### Effects of catechol on COX-1 and COX-2 enzyme activity

The direct effect of catechol on COX-1 and COX-2 enzymatic activities was measured using the COX inhibitor screening assay according to the manufacturer's instructions [6]. In brief, catechol (final 1–50 µM) was incubated with COX-1 or COX-2 in a reaction buffer for 10 min. Thereafter, AA was added as a substrate and incubated for an additional 2 min. This was followed by the addition of 0.1 N HCl and saturated stannous fluoride solution to stop the reaction. COX-1- or COX-2-mediated PGE_2_ production was then measured using the Cayman ELISA kits.

### Fluorimetric assay of ROS production in platelets and PMNs

Platelet ROS production was determined by measuring the platelet 2′,7′-dichlorofluorescein (DCF) fluorescence as described by Iuliano et al. [20] with slight modification. Briefly, washed platelets (5×10^8^/mL) were preincubated with 10 µM 2′,7′-diacethyl- chlorofluorescein (DCFH-DA) at 37°C for 30 min. After centrifugation and washing, 200 µL of platelets (5×10^8^/mL) were added to each well of a 96-well plate containing various concentrations of catechol (final concentrations of 1–50 µM). Then AA (final 100 µM), and the emitted DCF fluorescence was measured immediately by a Gemini XPS microplate spectrofluorometer (Molecular Devices Corporation, Sunnyvale, CA, USA) at an excitation of 485 nm and emission of 535 nm for 15 min. The fluorescence of DCF-labeled platelets without exposure to AA was used as a reference of basal level ROS production.

Human polymorphonuclear leukocytes (PMNs) were isolated as previousy described [16], by the approval of Ethics Committee, National Taiwan University Hospital. Briefly, heparinized blood was mixed with 3% dextran at a ratio of 1∶1 (v/v) in a 50 mL conical tube and placed upright for 20–25 min at room temperature. Then, 10 mL of Ficoll-Hypaque lymphocyte separation medium (LSM1077-PAA Labs, Pasching, Austria) was added, mixed and density-gradient centrifuged at 400×g for 40 min at 4°C. After hypotonic lysis of the erythrocytes, the isolated PMNs (final 4×10^6^ cells/mL) were counted and resuspended in 1× Hanks' balanced salt solution (HBSS) containing 10 µM DCFH-DA for 40 min. Cells were washed with HBSS prior to the addition of phorbol myristate acetate (PMA, final 1 µM) with and without catechol (1–100 µM) pretreatment. The emitted DCF fluorescence was measured using a Gemini XPS microplate spectrofluorometer at an excitation of 485 nm and emission of 535 nm for 20 min. SOD and NAC was used to prevent PMA-induced ROS production by PMNs.

### Effect of catechol on AA-induced ERK and p38 phosphorylation in platelets

The prepared platelets (1×10^9^ platelets/mL) were resuspended in Tyrode's solution containing 1 mM CaCl_2_ and 0.35% BSA, and 0.5 mL of the platelet suspension was placed into an aggregometer with constant stirring. Various amounts of catechol were added and preincubated for 3 min followed by addition of AA (100 µM) for 5 min. After addition of 0.5 mL of Laemmli sample buffer (75 mM Tris-HCl, 2% SDS, 15% glycerol, 3% 2-mercaptoethanol, pH 6.8) to terminate the reaction and for platelet lysis, cell lysates were boiled for 5 min, and 40 µL of cell lysates were subjected to 8% SDS-PAGE. After transfer to a PVDF membrane, blots were probed using anti-GAPDH, anti-p-ERK and anti-p-p38 antibodies as described previously [16]. For some western blot results, Image J software was used for quantitative analysis. The results were expressed as folds of control (as 1).

### *Ex vivo* platelet aggregation of platelet-rich plasma (PRP)

After intravenous injection of catechol (0.5–5 µmole/mouse), blood was drawn from each mouse by heart puncture [21]. Whole blood containing anticoagulant (3.8% sodium citrate in 9∶1 (v/v)) was placed in an Eppendorf tube and centrifuged at 60×g for 4 min to prepare PRP. AA-induced *ex vivo* platelet aggregability of PRP was measured with an aggregometer as previously described [6], [16], [21].

### Effect of catechol on IL-1β-induced PGE2 production and COX-2 expression of dental pulp cells

PGE_2_ level of dental pulp and its induction by IL-1β is crucial to the pathogenesis of pulpitis and pulpal pain [22], [23]. To know the anti-inflammatory effect, we tested whether catechol may prevent IL-1β-induced COX-2 expression and PGE_2_ production of pulp cells in this study. Human dental pulp cells were cultured as described previously [14]. For PGE_2_ analysis, 1×10^5^ pulp cells were inoculated onto 24-well culture plates. After 24 h, medium was changed, and catechol (50 and 100 µM) was added for a 30 min pretreatment followed by addition of IL-1β (5 ng/mL) for an additional 24 h. Culture medium was collected for PGE_2_ analysis by ELISA. Viability of pulp cells was evaluated by MTT assay as previously [16], because pulp cells are attached to culture wells that is different from platelets floating in the buffer. For analysis of COX-2 expression, 1.5×10^6^ pulp cells were inoculated onto 10-cm culture dishes. After 24 h, medium was changed, and catechol (50 and 100 µM) was added for a 30 min pretreatment, followed by addition of IL-1β (5 ng/mL) for an additional 24 h. Cell lysate of cultured pulp cells were prepared and subjected to western blotting as above (section 2.7.) except that anti-GAPDH and anti-COX-2 antibodies were used.

### Statistical analysis

Three or more independent experiments were performed. Results were expressed as percentage of inhibition on AA-induced platelet aggregation and TXB_2_ synthesis. One-way ANOVA and post-hoc Tukey test were used for statistical analysis.

## Results

### Effects of catechol on platelet aggregation

**Figure 1:**
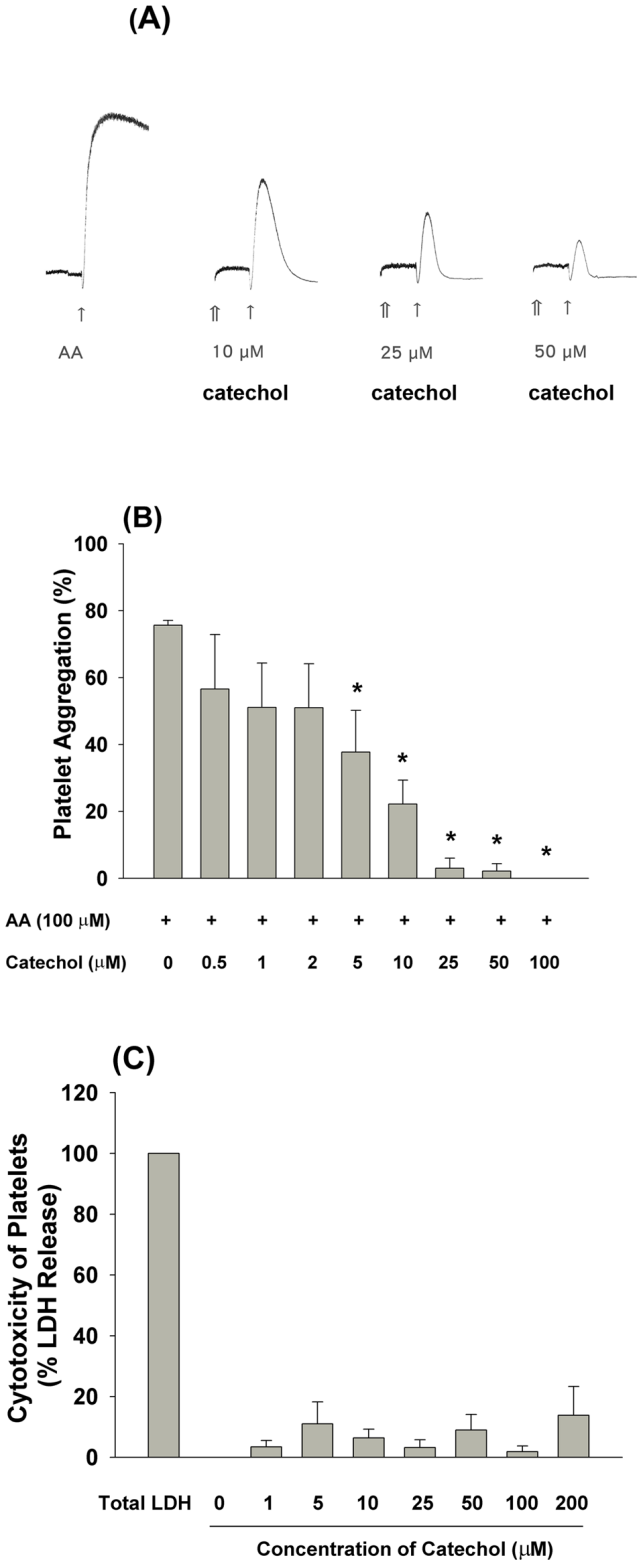
Inhibition of AA-induced platelet aggregation by different concentrations of catechol. (A) One representative picture of AA-induced platelet aggregation and its inhibition by catechol was shown. (B) Quantitative analysis of AA (100 µM)-induced platelet aggregation and its inhibition by catechol (0.5–100 µM). Results were expressed as % of platelet aggregation (n = 5–8). (C) Cytotoxicity of catechol to platelets. Results were expressed as % of LDH release (n = 3). ↑indicates the addition of AA, and ⇑ indicates the addition of catechol. *indicates significant difference when compared with AA-treated group (P<0.05).

The effects of catechol on platelet aggregation were first analyzed. As shown in **Figure 1A**, catechol suppressed AA-induced platelet aggregation at concentrations ≥10 µM. Quantitative analysis revealed that 5, 10 and 25 µM catechol inhibited AA-induced platelet aggregation by 50%, 71% and 94%, respectively (***p*** **<0.05;** **Figure 1B**), and 100 µM catechol completely inhibited AA-induced platelet aggregation (***p*** **<0.05;** **Figure 1B**). However, it did not significantly induce platelet cytoxicity at concentrations of 1–200 µM as determined by LDH release (**Figure 1C**).

### Inhibition of COX-1 and COX-2 activities by catechol

**Figure 2:**
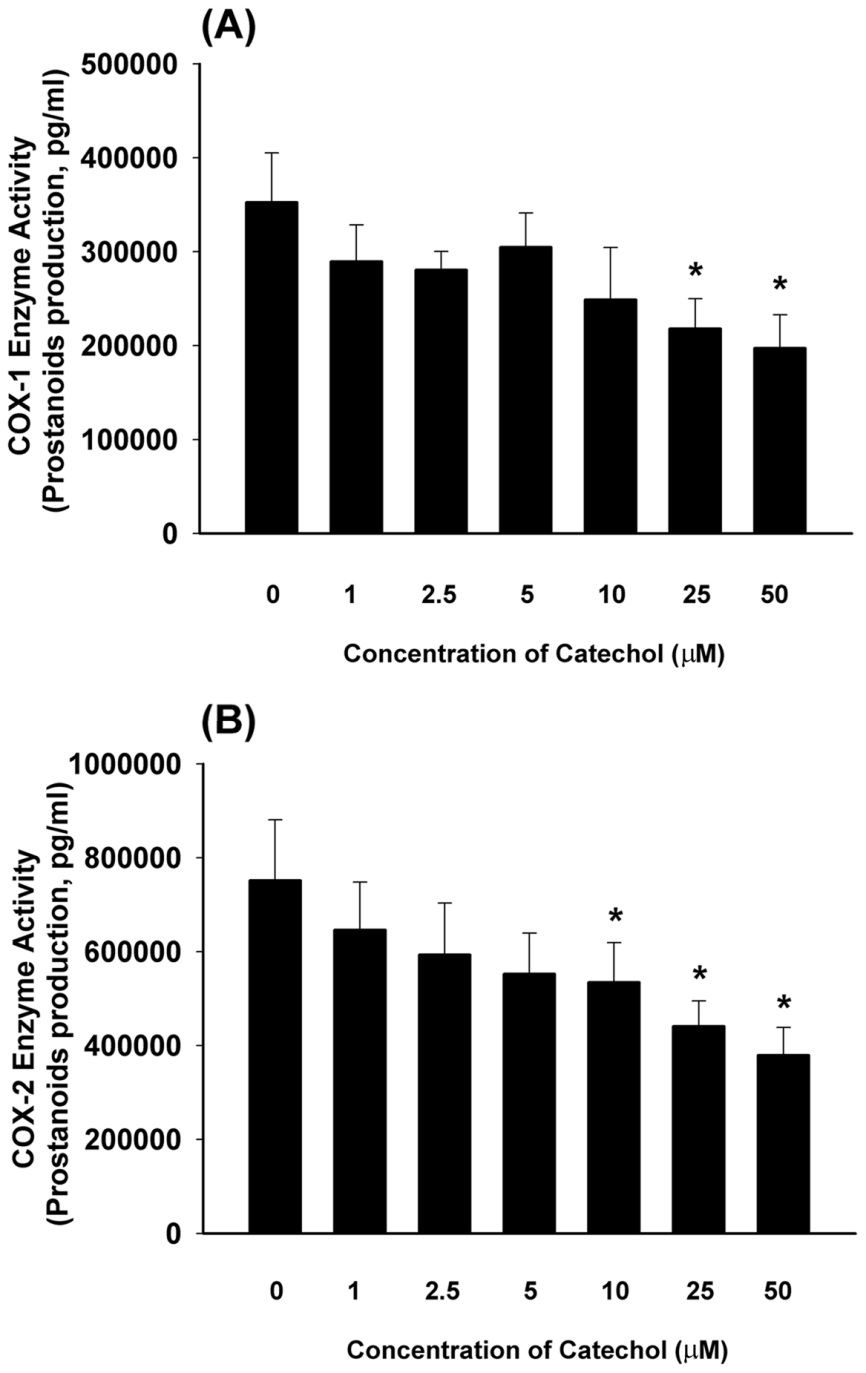
Effect of different concentrations of catechol on the (A) COX-1 enzyme activity (n = 6), and (B) COX-2 enzyme activity (n = 6). Results were expressed as COX-1 and COX-2 enzyme activity as indicated by PGE_2_ production (pg/mL). *indicated statistically significant difference when compared with solvent (DMSO) control group (0) (p<0.05).

The anti-platelet effect of catechol can be partly explained by its inhibition of COX-1 activity. As shown in **Figure 2A**, catechol inhibited COX-1 activity by 29%, 38% and 44% at concentrations of 10, 25 and 50 µM, respectively (***p*** **<0.05**;). Accordingly, catechol also inhibited COX-2 enzymatic activity by 29%, 42% and 50% at concentrations of 10, 25 and 50 µM, respectively (***p*** **<0.05;** **Figure 2B**).

### Effect of catechol on AA-induced platelet TXB_2_ production and U46619-induced platelet aggregation

**Figure 3:**
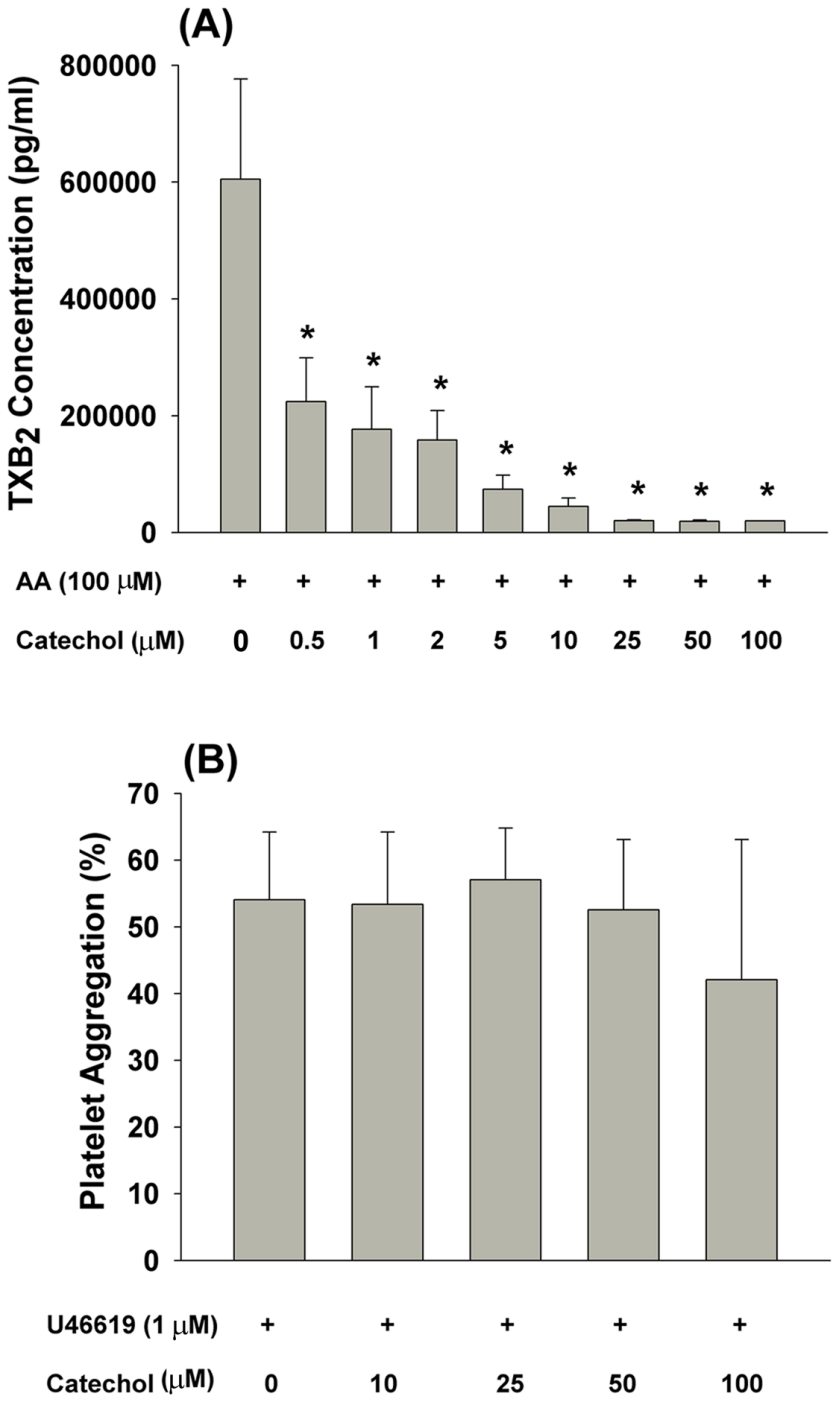
Effect of catechol on AA-induced TXB_2_ production in platelets, (A). Results were expressed as TXB_2_ production (n = 5). *denotes statistically significant difference when compared with the group that treated by solvent (DMSO) plus AA, (B) Effect of catechol (10–100 µM) on U46619 (1 µM)-induced platelet aggregation (n = 3).

We next assessed the effects of catechol on AA-induced TXB_2_ production by platelets. As shown in **Figure 3A**, catechol suppressed AA-induced TXB_2_ production by 64% and 88% at concentrations of 0.5 and 5 µM, respectively (***p*** **<0.05**).

U46619 (1 µM), a TXA2 receptor agonist, can stimulate platelet aggregation. This effect was not significantly altered by catechol at concentrations ranging from 10–100 µM (**Figure 3B**).

### Effects of catechol on AA-induced ROS production by platelets

**Figure 4:**
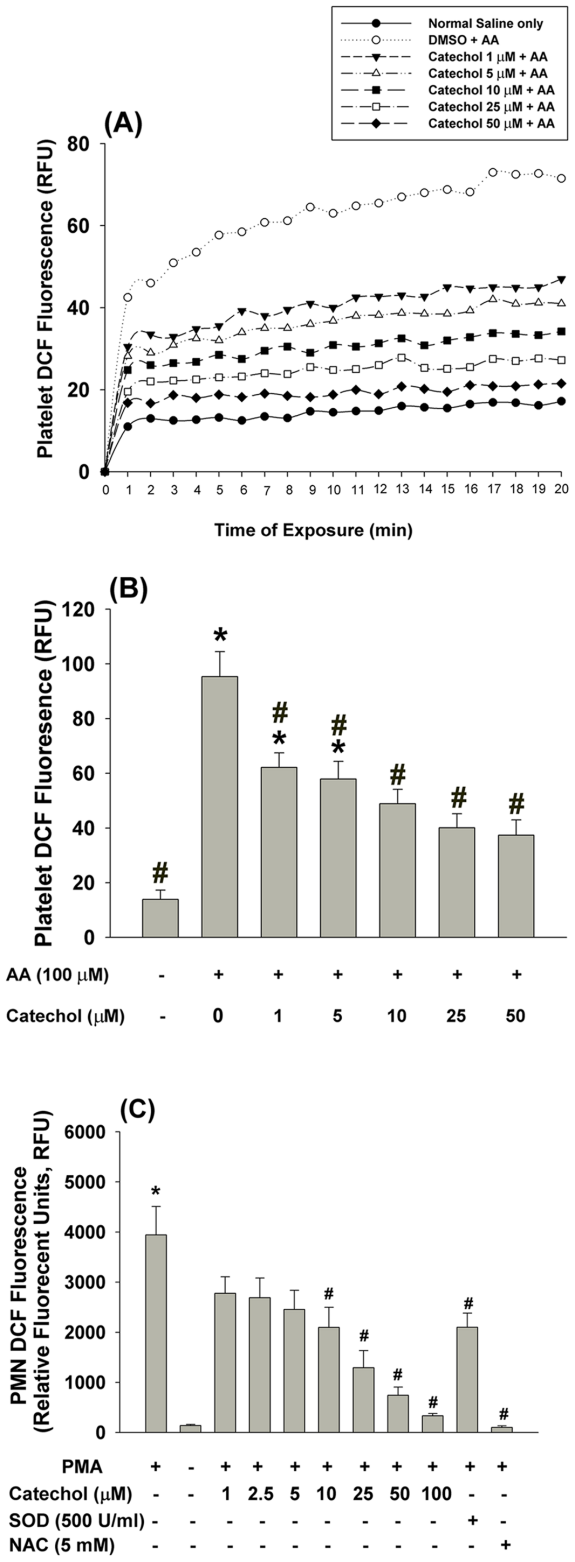
Effect of catechol on AA-induced ROS production in platelets, (A). The DCF fluorescence of platelets after exposure to AA with/without catechol was measured. One representative histogram of DCF fluorescence was shown. Results were expressed as relative fluorescent unit (RLU). (B) Quantitative DCF fluorescence of platelets after exposure to AA with/without pretreatment by solvent control (DMSO) or catechol. *denotes significant difference (p<0.05) when compared with normal saline treated group (NC). #denotes statistically significant difference when compared with solvent (DMSO)+AA group (n = 4). (C) Catechol also prevented PMA-induced ROS production in isolated human PMN (n = 6). *denotes significant difference (p<0.05) when compared with control group. #denotes statistically significant difference when compared with vehicle + PMA group

Exposure of platelets to AA rapidly stimulated cellular ROS production as analyzed by an increase in DCF fluorescence. Pre-incubation with catechol attenuated AA-induced DCF fluorescence in platelets (**Figure 4A**). At concentrations of 1 and 10 µM, catechol effectively suppressed AA-induced ROS production by 35% and 49% (***p*** **<0.05;** **Figure 4B**). Moreover, the PMA-induced ROS production in isolated human PMNs as indicated by an increase in DCF fluorescence. This was inhibited by SOD (500 U/mL), N-acetyl-L-cysteine (NAC, 5 mM) as well as catechol (10–100 µM) (***p*** **<0.05;** **Figure 4C**).

### Effects of catechol on AA-induced p38 and ERK phosphorylation in platelets

**Figure 5:**
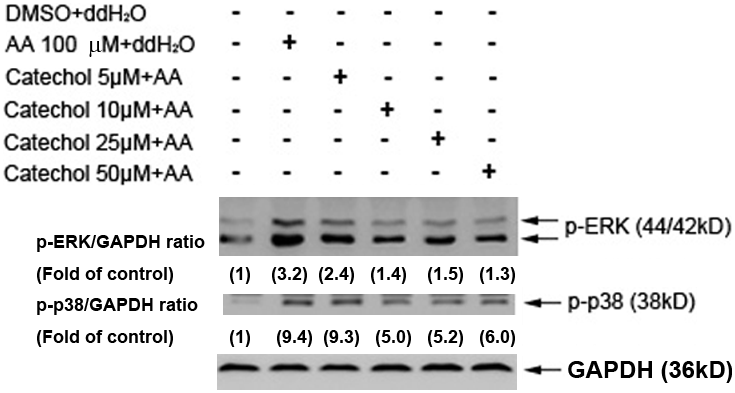
Effect of catechol on AA-induced p38 and ERK phosphrylation in platelets. One representative western blotting picture was shown. Expression of GAPDH was used as control. Quantitative results of p-ERK/GAPDH and p-p38/GAPDH ratio were measured by Image J analysis and expressed as fold of control (the value in parenthesis).

Mitogen-activated protein kinases (MAPKs), such as ERK and p38, are important for the regulation of platelet aggregation. To determine whether the anti-platelet effect of catechol is due to its attenuation of MAPK signaling, we incubated platelets in AA and found that it rapidly stimulated ERK and p38 phosphorylation (**Figure 5**). However, pre-incubation of platelets with catechol (5–50 µM) attenuated AA-induced ERK and p38 phosphorylation as analyzed by Western blot analysis. Quantitatively, AA stimulated ERK and p38 phosphorylation to 3.2–fold and 9.4–fold of control, respectively (**Figure 5**). Catechol (10–50 µM) decreased the AA-induced p-ERK and p-p38 expression to 1.3–1.5 fold and 5.0–6.0 fold of control, respectively (**Figure 5**).

### Effects of catechol on *ex vivo* platelet aggregation induced by AA

**Figure 6:**
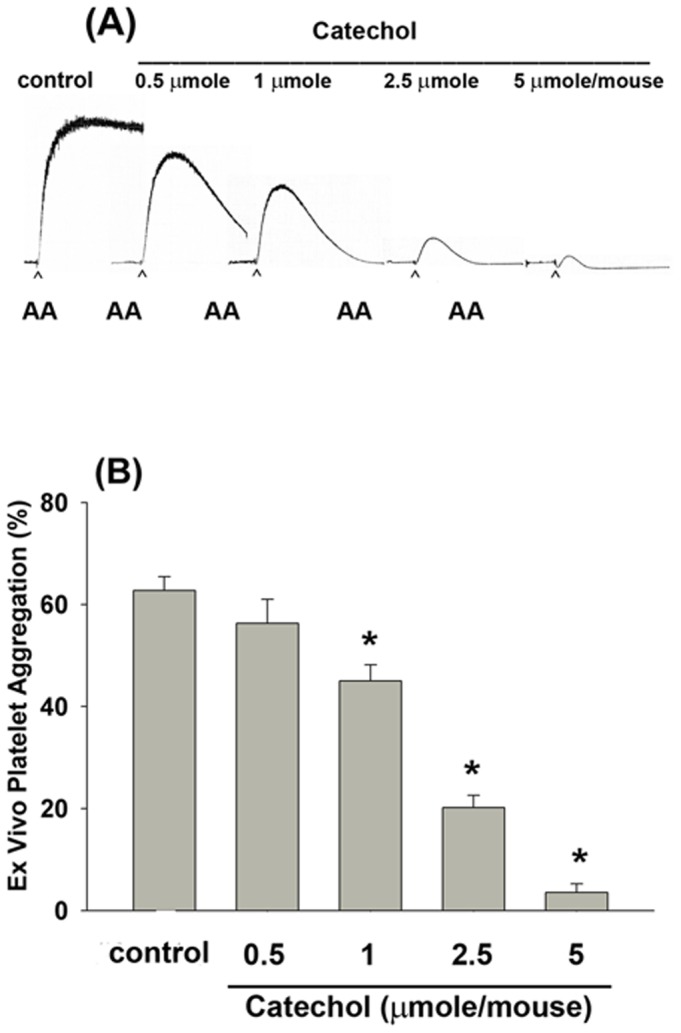
*Ex vivo* platelet aggregation by AA and its inhibition by administration of catechol. (A) Intravenous administration of catechol (0.5–5 µmole/mouse) inhibited the AA-induced platelet aggregation *ex vivo*. One representative picture of platelet aggregation was shown with more similar results. (B) Inhibition of AA-induced platelet aggregation *ex vivo* by intravenous administration of different amounts of catechol (0.5–5 µmole/mouse) into mice. (n = 7). *denotes significant difference when compared with solvent control group (p<0.05).

Intravenous administration of catechol suppressed AA-induced platelet aggregation *ex vivo* at concentrations higher than 1 µmole/mouse (**Figure 6A**). Specifically, 2.5 and 5 µmole catechol/mouse significantly inhibited *ex vivo* AA-induced platelet aggregation by 68% and 94%, respectively (***p*** **<0.05;** **Figure 6B**).

### Effects of catechol on the growth, PGE2 production and COX-2 expression of dental pulp cells

**Figure 7:**
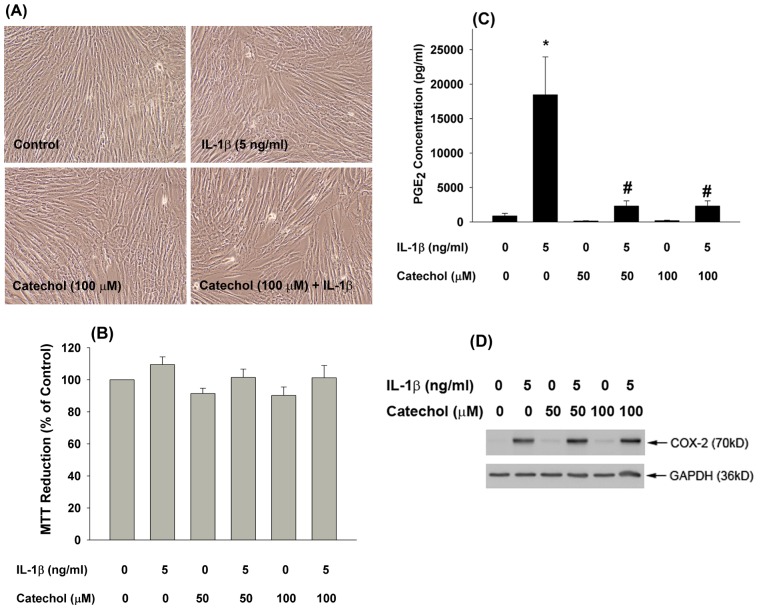
Catechol inhibited the IL-1β-induced PGE_2_ production of pulp cells. Pulp cells were pretreated with catechol with/without further co-incubation by IL-1β for 24-hours. (A) morphology of control fibroblasts, pulp fibroblasts exposed to IL-1β for 24-hours, Pulp fibroblasts incubated with 0.1 mM catechol for 24-hours, and Pulp fibroblasts exposure to 0.1 mM catechol with IL-1β for 24-hours, (B) Viable cells were estimated by MTT assay. MTT reduction was shown as % of control (Mean ± SE) (n = 8). *denotes significant difference (p<0.05) when compared with solvent control group. (C) PGE_2_ level in the cultured medium was measured and the PGE_2_ concentration was shown as pg/ml (Mean ± SE) (n = 7). *denotes significant difference when compared with solvent control group; #denotes significant difference (p<0.05) when compared with IL-1β solely group. (D) COX-2 expression of pulp cells after exposure to IL-1β with/without pretreatment and co-incubation by catechol. One representative western blotting result was shown.

Catechol inhibited COX-1 and COX-2 activities (**Figures 2A and 2B**). Because we have found that IL-1β stimulates COX-2 expression and PGE_2_ production by pulp cells [23], we further analyzed whether catechol may have anti-inflammatory effect and could inhibit IL-1β-induced PGE_2_ production of pulp cells. Exposure to 50 and 100 µM catechol with/without IL-1β showed little effect on pulp cell morphology as compared with control pulp cells (**Figure 7A**), and minimal cytotoxicity with 50 and 100 µM catechol was observed (**Figure 7B**). However, catechol (50 and 100 µM) almost completely inhibited the IL-1β-induced PGE_2_ production by pulp cells (**Figure 7C**). Western blotting analysis further found that catechol (50 and 100 µM) by itself slightly stimulated COX-2 protein expression of pulp cells. In addition, pretreatment by catechol and co-incubation with IL-1β showed no marked stimulation and even enhanced the IL-1β-induced COX-2 expression (**Figure 7D**), suggesting a possible compensatory response.

## Discussion

Catechol is present in a number of food products and cosmetic ingrediens. It can be rapily absorbed by oral, dermal and inhalaton of exposure. Skin application of catechol for 30 min results in absorption of 0.4% of applied dose through human skin and a 24-h application of 4% catechol results in 53% absorption from skin, where it can be metabolized to reactive benzoquinone metabolites or other toxic quinone species by myeloperoxidase [24], [25]. It is also a metabolite of benzene and toluene [4]. In this study, catechol exhibits anti-platelet effects at low concentrations, suggesting its potential role in the prevention of cardiovascular diseases at low levels. Consistently, catechol, resorcinol (1,3-benzenediol) and hydroquinone have been previously shown to suppress AA-induced rabbit platelet aggregation although with varying levels of potency (about µM concentrations) [12]. Catechol also inhibits collagen-induced platelet aggregation at concentration of 100 µM, and a catechol group of phenolics has been suggested to responsible for its anti-platelet effect on collagen-induced platelet aggregation [13], [26]. In the present study, catechol significantly inhibits AA-induced platelet aggregation even at concentrations higher than 5 µM, which is in general agreement with the results described by Kitagawa et al. [12]. It is well known that platelet dysfunction plays a critical role in the pathogenesis of cardiovascular diseases [9]–[11]; therefore, exposure to catechol or consumption of catechol-containing products at suitable amounts may potentially prevent cardiovascular diseases via its anti-platelet effect.

AA-induced platelet aggregation is mediated by COX-1 activation, TXA_2_ production and subsequent activation of thromboxane receptors and the downstream signaling to induce full platelet aggregation. In this stdy, the anti-platelet effect of catechol may be partly due to its inhibition of COX-1 enzymatic activity given that platelets only express COX-1 and the binding and inhibition of catechol on COX and phospholipase A_2_ (PLA_2_) has been reported [27], [28]. Consistently, catechol almost completely inhibited AA-induced platelet production of TXA_2_, which interacts with platelet thromboxane receptor and induces full platelet aggregation. But catechol showed only partial inhibition on COX activity. This indicates that catechol possibly also affects other critical molecules such as ROS and PLA_2_ as mentioned previously [28]. As a stable TXA_2_ metabolite, TXB_2_ was obviously inhibited by catechol in AA-stimulated platelets. Binding of TXA_2_ to platelet TXA_2_ receptors may further induce downstream signaling for platelet aggregation [29]. Platelet TXA_2_ production is mediated mainly through metabolism of AA by COX-1 to produce TXA_2_, that may bind and activate thromboxane receptor to induce further full platelet activation. The TXA_2_ receptor agonist, U46619, was shown to stimulate marked platelet aggregation. However, catechol showed little inhibitory effect on U46619-induced platelet aggregation, suggesting that the anti-platelet effect of catechol is associated with COX-1 inhibition and that it cannot suppress platelet aggregation signaling downstream of thromboxane receptors.

The anti-platelet effect of catechol was not due to cytotoxicity as no obvious changes in platelet extracellular and intracellular LDH levels were noted after exposure to catechol. Catechol is not toxic to glioblastoma cells at concentrations below 0.2 mM [30]; however, it induces a 44% increase in esophageal cell death at 35 µM [4], suggesting that it exhibits differential cytotoxicity that is cell type-dependent.

ROS may regulate many physiological and pathological processes in the pulmonary and cardiovascular systems [31], [32]. ROS and redox changes also critically modulate platelet function [20], [33]. In this study, we found that AA stimulated ROS production in platelets, which was suppressed by catechol. Catechol further inhibited PMA-induced ROS production in PMNs, which is suggestive of a potential role as an anti-oxidant and anti-inflammatory mediator. Thus, the anti-platelet and anti-inflammatory effects of catechol are possibly related to its scavenging ROS or attenuation of ROS production in platelets. Consistently, the catechol group within the B-ring of flavonoids is responsible for their anti-oxidant effects toward PMA-induced oxidative burst [34] and 1,1-diphenyl-2-picrylhydrazyl free radicals [35]). However, at higher concentrations (150–600 µM), catechol is toxic to cultured Muller cells possibly via ROS production [36]. Therefore, more *in vitro* and *in vivo* studies are necessary to further clarify the beneficial and detrimental effects of catechol exposure.

ERK and p38 MAPKs play an important role in the regulation of platelet granule secretion, ATP release and platelet aggregation as well as the stimulation of myosin light chain to stimulate blood clot retraction [37], [38]. Some anti-platelet agents, including caffeic acid phenethyl ester, simvastatin and davallialactone, suppress platelet activation via inhibition of p38 and ERK [38]–[40]. Inhibition of both ERK and p38 phosphorylation is required to suppress AA-induced platelet aggregation [41]. In this study, we found that catechol effectively prevented AA-induced ERK and p38 MAPK phosphorylation, which is crucial for platelet AA release and TXA_2_ production [39]. At comparable concentrations, catechol obviously inhibited AA-induced platelet aggregation and TXA_2_ production in the present study, suggesting that the inhibitory effect of catechol on platelet TXA_2_ production is possibly mediated by blocking p38 and ERK signaling or other upstream signaling molecules such as phospholipase C (PLC), protein kinase C, glycoproein αII_b_β_3_, proline-rich tyrosine kinase 2 [42]–[44], and that it may have therapeutic potential as an anti-platelet agent.

In the present study, intravenous administration of catechol to mice inhibited *ex vivo* platelet aggregation, indicating that catechol may have anti-platelet effects even in the presence of plasma. Because catechol inhibits both COX-1 and COX-2 activities, we further analyzed the effects of catechol on IL-1β-induced PGE_2_ production of oral fibroblasts, especially given that it exhibits anti-microbial and anti-inflammatory activities by suppression of leukotriene B_4_ production in rat peritoneal exudate cells [45]. Therefore, catechol may increase the PGE_2_/leukotriene B_4_ ratio in PMNs. These phenols also inhibit PGE_2_ production in whole blood. Low concentrations of catechol stimulated PGE_2_ formation in PMNs; however, a repression was noted at high concentrations [46]. Vascular wall inflammation is related to platelet activation and constitutes a major pathogenic event in atherosclerosis [10]. Stimulation of COX-2 expression and PGE_2_ production is crucial for tissue inflammation, pain response and cardiovascular disease [23], [47], [48]. Dental pulp is a specialized connective tissue popularly suffered from inflammatory and pain response, where IL-1β and PGE_2_ are involved [22]. Recently, we observed the stimulation of PGE_2_ by IL-1β and possible activation of pulp cells via prostaglandin EP receptors, and suppression of IL-1β-induced PGE_2_ production in pulpal fibroblasts by plant phenolic was noted [16], [23]. In this study, catechol also attenuated the IL-1β-induced PGE_2_ production, but no COX-2 expression of pulp cells. These results support the anti-inflammatory effect of catechol. The inhibition of IL-1β-induced PGE_2_ production of pulp cells by catechol is possibly due to its direct interaction with COX-2 and inhibition of COX-2 enzyme activities, but not due to its suppression of COX-2 protein expression.

## Conclusions

Catechol exhibits anti-platelet and anti-inflammatory effects. The anti-platelet effects of catechol are related to its inhibition of platelet COX activity, TXA_2_ production, ROS production as well as ERK/p38 signaling. The anti-platelet effect of catechol was confirmed using an *ex vivo* study. Catechol further suppresses PMA-induced ROS production in PMNs, which is suggestive of possible anti-inflammatory properties. Catechol also exhibits anti-inflammatory effects via inhibition of COX-2 activity and IL-1β-induced prostaglandin production. Catechol showed little cytotoxicity at concentrations below 0.1 mM. Taken together, exposure to catechol or suitable consumption of catechol-containing food/nutritional supplements, such as marine algae tablets or plants, may be beneficial to cardiovascular health. Delineation of its full pharmacological potential requires further investigation.
